# Factors Affecting Adaptability of Cryptocurrency: An Application of Technology Acceptance Model

**DOI:** 10.3389/fpsyg.2022.903473

**Published:** 2022-06-03

**Authors:** Nadia Sagheer, Kanwal Iqbal Khan, Samar Fahd, Shahid Mahmood, Tayyiba Rashid, Hassan Jamil

**Affiliations:** ^1^Institute of Business and Management, University of Engineering and Technology, Lahore, Pakistan; ^2^Department of Applied Psychology, The Islamia University of Bahawalpur, Bahawalpur, Pakistan; ^3^Institute of Business Management and Administrative Sciences, The Islamia University of Bahawalpur, Bahawalpur, Pakistan; ^4^Institute of Quality and Technology Management, University of the Punjab, Lahore, Pakistan; ^5^UNSW Institute for Cyber Security, Australian Defence Force Academy, University of New South Wales, Canberra, ACT, Australia

**Keywords:** government support, perceived usefulness, perceived ease of use, behavioral intention, perceived risk, technology awareness, technology acceptance model

## Abstract

Cryptocurrency has revolutionized the economic system of the world. It provides a new and innovative means of exchange that has speedily invaded the financial market trends and changed the traditional cash world. However, consumers have low acceptability for blockchain-based cryptocurrency due to increasing online scams and the absence of a regulatory framework. There is also a misconception about its usage on many platforms, which has created a clear gap in the literature to address this issue. Therefore, the current study intends to investigate the effect of technology awareness on the behavioral intention of crypto users through perceived factors (usefulness, ease of use, risk). It also empirically examines the moderating role of government support on these indirect paths. The underlying framework is investigated by surveying 333 respondents from the Z generation. Results revealed that perceived factors (usefulness, ease of use, risk) mediate the relationship between technology awareness and behavioral intention. Furthermore, government support strengthens the indirect relationship of technology awareness on behavioral intention through technology acceptance determinants, such that the effect of technology awareness on behavioral intention through perceived factors (usefulness, ease of use, risk) is more assertive when government support is high. The findings will provide a new dimension to different financial bodies implementing monetary policy and highlight the need to adopt innovative digital technologies in Pakistan.

## Introduction

The digital economy is quickly rising and developing worldwide, causing all market players to experience significant changes in their operations (Nasir et al., 2021). It not only modernizes payment methods but also has the potential to influence the future of virtual currencies. According to the statistics, the total amount of transactions in the digital payments category is estimated at US$ 6.752,388 million in 2021, which is expected to grow by 12.24% every year (Patel and Shrimali, 2021). Cryptocurrency is also one of the famous types of digital currency due to its unique features of decentralization, fast mode of exchange, and being free from geographical borders (Zafar et al., 2021). World Bank considers it a non-fiat digital currency without any intrinsic value, which is not backed by any assets and cannot be claimed as a liability from an economic intermediary (Natarajan et al., 2017). They are based on blockchain technology and employ cryptographic techniques.

By using blockchain-based technology, international businesses are being enhanced and are shifting toward cryptocurrency for monetary transactions (Han et al., 2021). Many sectors like e-commerce are flourishing today, but they often face issues like fraud, commission costs, restricted buyer-seller communications, and abuse of personal data (Frik and Mittone, 2019). However, using cryptocurrency in payment and smart contracts alleviates these problems by increasing security and transparency (Ismanto et al., 2019). That is why they are gaining popularity among people for managing current payment systems by providing a new understanding of money and carrying out online transactions without involving a third party (Johar et al., 2021). Although, it is a crucial investment vehicle that cannot be neglected in the current digitized era (Uematsu and Tanaka, 2019). However, its future will depend on how investors utilize it and how financial institutions regulate it (Berentsen and Schär, 2018).

Previously, researchers believed that technology awareness could increase the probability of accepting new advancements (Irfan et al., 2022). Other factors like lack of knowledge of digital handling and government policies are also causing an obstacle to its adoption (Putri et al., 2021). Many specialists in blockchain technology are debating about its complicated characteristics and unique structure, which is not simple to understand by the consumers (Doblas, 2019). According to a survey, non-users of cryptocurrency felt unable to utilize it without advanced technological knowledge (Dryall, 2018). In addition to that, financial literacy can restrict its adaptability (Arias-Oliva et al., 2019). Previous studies stated that perceived factors (usefulness, trust, ease of use, experience), government regulations, and support play a significant role in influencing the intention of the user to adopt cryptocurrency (Wu and Tran, 2018).

Research on the uses and gratification focuses on how users feel delighted while using unique and innovative technologies in daily life for comfort and ease (Shafieizadeh et al., 2021). In contrast, perceived usefulness depicts the feeling of the consumers when they consider that their selected service will be improved by the application of technology (Hileman and Rauchs, 2017). Many researchers have linked perceived factors (usefulness and ease of use) with the behavioral intention of consumers to adopt new technology (Saadé and Bahli, 2005; Daud et al., 2018). In the case of cryptocurrency, studies revealed that perceived behavioral factors are the most critical factors in deciding whether to utilize them for electronic payments (Schaupp and Festa, 2018). Individuals who believe cryptocurrencies are simple to use are more inclined toward them. Shahzad et al. (2018) found that perceived factors substantially impacted the intention to embrace cryptocurrency. That is why it is essential to consider these factors for future studies (Mendoza-Tello et al., 2018).

The perceived risk is the outcome of a choice representing the difference between the final results and the degree to which innovation is implemented (San Martín and Camarero, 2009). People are afraid of embracing new technology for various reasons, while the most common is the level of uncertainty (Lim, 2003; Mizanur and Sloan, 2017). Jalan et al. (2021) defined risk as a knowledge of the degree of insecurity used to consider investors’ or customers’ decisions to adopt a technology (Slovic et al., 1982; Anser et al., 2020). Moreover, perceived risk was a crucial factor in using and adopting technology. Therefore, security and risk are also considered concerning aspects for customers to adapt to new technology (Granić and Marangunić, 2019) and affect the consumers’ behavioral intention (Mutahar et al., 2018). Cryptocurrencies are a new financial technology that has the potential to be risky. Legislation and legality are the primary concerns from the user’s standpoint to adopting this technological innovation in the monetary system (Albayati et al., 2020). But, the moderating role of government support on the relationship between technology acceptance variables and behavioral intention still requires more attention (Zafar et al., 2021).

The theory of planned behavior related to technology usage also highlights the role of an awareness-acceptability framework (Hung et al., 2006). Researchers are still modifying and evaluating the suitable theoretical framework for the new technology acceptance model (TAM) to identify the most influential determinants (Hamilton, 2020). That is why the current study analyzes the relationship between technology awareness and behavioral intention of crypto users through perceived factors (usefulness, ease of use, risk). It also investigates the moderated role of government support in these indirect relationships in the context of Pakistan because Pakistan is an emerging market for this blockchain technology. Still, there has been low technology awareness among the masses for these digital innovations. It is still at an infant stage among citizens, causing the main hindrance in its adaptability (Rizvi et al., 2018).

The present study has provided significant insight into the development of blockchain technology as the medium of exchange in Pakistan. First, it strengthens the value of an existing body of knowledge related to technology awareness and the behavioral intention of consumers to adopt blockchain technology by confirming the significant role of perceived factors (usefulness, ease of use, risk). It helps in evaluating the aspects that encourage consumers to adopt this technology. Second, it will add to a body of knowledge to the fast evolution in the transformation of financial set-ups, which strengthens the advancement in the ongoing monetary system that has been outmoded. Third, as cryptocurrency significantly affects the international economic system, the current study helps the existing resources get digitized and monetary channels to derive new models for the monetary exchange system. Lastly, its social significance includes its potential to use virtual currencies for transactions and investment purposes in the future. Industries are changing due to these new technologies, and enterprises can advertise their plans and business models according to the actual demands of financial markets.

## Theories and Literature Review

### Theories for Technology Acceptance of Cryptocurrency

The primary objective of the study is to identify the behavioral intention of customers regarding the adoption of cryptocurrency. Previous studies consider the predictors of the TAM for describing and forecasting how a new system will be utilized (Zhao et al., 2010). However, the TAM cannot be the only tool to evaluate cryptocurrency’s acceptance, so other theories are also considered (Berentsen and Schär, 2018). To illustrate the acceptability and an adopter’s willingness to use cryptocurrency, the theory of planned behavior and reasoned action can be integrated, which was taken from earlier work on technology usage by LaCaille (2020). These are conceptual theories that provide a theoretical framework for interpreting human behavioral intention in specific situations (LaCaille, 2020). They support the TAM and help to understand and identify technological usage (Khan et al., 2020).

These theories presented a TAM to understand better and forecast human behavior toward using technology, which has been adopted by many technology adaptation studies (Johar et al., 2021). Furthermore, users’ technology awareness can be an essential tool to fully comprehend and understand this model’s basic components: perceived ease of use and perceived usefulness to knowing well about the customer’s behavioral intention toward adopting new technology (Venkatesh, 2000). Behavioral intention is the best predictor of action (e.g., toward an information system or system utilization) and intentions toward adopting new technologies (Fagnant and Kockelman, 2015). Technology acceptance model determinants help consumers make the best decisions regarding technology acceptance by guiding them through the processes and procedures (Shahzad et al., 2021).

### Technology Awareness and Behavioral Intention

Behavioral intention quantifies customers’ willingness to embrace financial transactions in the context of cryptocurrency (Almuraqab, 2020). Technology awareness and behavioral intention are considered the most critical factors determining technology adoption (Igbaria et al., 1994). In the case of cryptocurrencies, it is a very widely classified topic because of their growing popularity (Uematsu and Tanaka, 2019). However, it has gained much popularity and has become a point of discussion for many new mediums, especially monetary implementations. But acceptability and usage of this currency are very low from the expected level (Hamilton, 2020). This condition leads to a search for why customers are hesitant to use virtual currencies and why most people rely on traditional payment methods even though they are riskier (Almuraqab, 2020).

Cryptocurrencies’ acceptance depends on the behavioral intention of consumers or investors. It includes their belief that this technology is helpful for them to enhance their performance, is easy to adapt, and prevails low-risk factors (Khan et al., 2021). The current status of its adoption is not secure by government regulations, so it can negatively impact its acceptance (Shahzad et al., 2018). Furthermore, the previous study gave an argument that the leading causes behind the low rate of adoption of cryptocurrency include problems for traders in understanding its rate fluctuations in the informal market, lack of liquidity of this currency, no financial principles, lack of government support, and high risks of security in this currency (Putri et al., 2021). These problems directly impacted the behavioral intentions and prevented them from becoming part of the new digital economy (Granić and Marangunić, 2019).

The awareness perception was first applied in an innovative diffusion theory (Fry and Cheah, 2016). Later, the innovative diffusion theory was applied to studies on implementing technology, confirming a positive impact of technology awareness on the behavioral intention of the consumer to adopt new technology (Alaeddin and Altounjy, 2018). The degree to which people are aware of and understand digital currency substantially impacts their willingness to use it (Treiblmaier and Sillaber, 2021). It is worth noting that users’ access to the available information about the benefits and potential risks and the strategies that are usually employed in adopting technology is classified as awareness (San Martín and Camarero, 2009). If the consumers believe that they have a knowledge and understanding of the key components, their perspectives about cryptocurrency and willingness to accept it will change (Ayedh et al., 2020).

Previous studies state that technology awareness and behavioral intention are strongly correlated and have a positive relationship (Barbu et al., 2021). Behavioral intention plays a significant role in accepting or rejecting any technology. If people have technology awareness, their behavior toward adopting that technology will be positive. Many researchers found behavioral measurements and their relationship with awareness of technological advancements correlate with each other (Yi et al., 2021). Behavioral intention quantifies customers’ willingness to embrace financial transactions in the context of cryptocurrency (Almuraqab, 2020). Technology awareness and behavioral intention are considered the most critical factors determining technology adoption (Igbaria et al., 1994).

### Mediating Role of Perceived Usefulness

The behavioral intention of people to accept or reject new innovative technology is influenced by two main determinants of the TAM. The first one is perceived usefulness which determines whether one can perform better using new technologies (Daud et al., 2018). It reflects the degree to which consumers believe that this technology will be helpful in boosting their performance (Venkatesh, 2000). The previous researcher examined the theoretical importance of perceived usefulness as the mediating role on behavioral intention (Robey, 1979). Because of technology awareness and perceived usefulness, users’ behavioral intention to adopt an application enhances. They believe that adopting new technology will improve their skills and abilities, which changes their behavioral intention to adopt new technology. Perceived usefulness is the technical quality connected to technology awareness and the behavioral intention of consumers (Granić and Marangunić, 2019).

The literature specifically on cryptocurrency and bitcoin considered perceived usefulness an essential factor impacting the intention to use virtual currencies as a medium of payments (Ferreira et al., 2018). It directly affects these relationships (Johar et al., 2021). According to another study on cryptocurrency, the core constructs for cryptocurrency adoption are based on the mediating role of perceived usefulness between technology awareness and consumers’ behavioral intention (Schaupp and Festa, 2018). The findings explain that perceived usefulness is a significant predictor that has previously been assimilated into studies related to cryptocurrency adoption (Kher et al., 2021). Therefore, hence it is hypothesized as:

*H_1_: Perceived usefulness mediates the relation between technology awareness and Behavioral intention to use cryptocurrency.*

### Mediating Role of Perceived Ease of Use

Perceived ease of use is how consumers believe that new technology is easy to use, easy to run, simple, responsive, and adaptable without much effort (Gould and Lewis, 1983). In other words, it depicts the beliefs of the people that adopting new technology will make their lives easy. It is the critical determinant of the TAM, and this construct has two direct formative relations with technology awareness and behavioral intention (Rizvi et al., 2018). Previously, many researchers used perceived ease of use to evaluate the behavioral intention of the users (Mutahar et al., 2018). Many studies found that perceived ease of use has a favorable influence on consumers’ intention to purchase (Blocki and Zhou, 2016). They stated that if there is no desired level of technological adoption or service acceptance, the system will not operate (Albayati et al., 2020).

Several researchers have discovered a strong correlation between perceived ease of use and intention to invest in new cryptocurrencies, supported by the theory of planned behavior (Alaeddin and Altounjy, 2018; Johar et al., 2021). Riquelme and Rios (2010) stated that perceived usefulness significantly influenced users’ perspectives and readiness to adopt technology when using complex information tools and techniques to complete financial transactions through digital devices (Mattke et al., 2021). Users are more inclined to adopt technological services that make their lives convenient, enjoyable, and easy to use (Riquelme and Rios, 2010). The previous analysis stated that perceived ease of use plays a mediating function between technology awareness and behavioral intention to use virtual currencies as a financial transaction (Chen and Aklikokou, 2020). Consumers believe that new technology saves time and effort (Hsu and Lu, 2004). Many researchers use perceived ease of use as the primary determinant to measure the behavioral intention of the consumer to adopt new technology, but very few got their desired results (McCloskey, 2007). The previous researcher has found that perceived ease of use can impact the behavioral intention of the consumer to adopt new technology and emphasized evaluating its mediating role between technology awareness and behavioral intention. Hence, it is hypothesized that:

*H_2_: Perceived ease of use mediates the relation between technology awareness and Behavioral intention to use cryptocurrency*.

### Mediating Role of Perceived Risk

In 1960, Raymond developed the notion of perceived risk to understand better customers’ behavior and the factors that impact their decisions (Mikhaylov, 2020). In perceiving a consumer’s behavioral intention, perceived risk represents a degree of uncertainty, and the negative repercussions of utilizing or purchasing a product (San Martín and Camarero, 2009). In the context of the purchase intention, perceived risk was considered a determining factor in evaluating a consumer’s behavioral intention (Shahzad et al., 2018). Perceived risk develops two direct relations with two primary constructs: technology awareness and consumers’ behavioral intention (Khan et al., 2021). The previous study explains that if consumers have less technology awareness and perceived risk, it will positively impact their behavioral intention, enhancing their willingness to adopt new technology (Chen and Aklikokou, 2020). A prior study describes how a person’s risk perception influences their behavioral intention (Shafieizadeh et al., 2021). Other conceptualizations combine perceived risks with the consumer’s perception of behavioral intention that influences technology adoption (Trudel et al., 2014).

Studies showed that consumers would adopt this technology if the perceived risk of using cryptocurrency were less. There are two frames for risk when it comes to cryptocurrency adoption. The first is that most people think cryptocurrency is riskier and related to speculative fraud and scams (Hileman and Rauchs, 2017). It happens due to the complexity of their operation and less knowledge of cryptography and computer science (Doblas, 2019). The second point is that many people are not aware of these new payment methods, which causes a feeling of unease and risks (Stocklmayer and Gilbert, 2002). Perceived risk is a deciding element in checking the behavioral intention of people toward the adaptability of cryptocurrency (Ayedh et al., 2020). For example, if a customer identifies a low degree of risk for cryptocurrency, confidence in adopting this technology will rise. According to previous research, higher technology awareness of digital currency can lower risk perception (Lim, 2003). If the perceived risk of cryptocurrency is reduced, it is feasible that users’ behavioral intention for this medium of exchange can improve even though they have technology awareness. It leads us to this relationship developed in the hypothesis.

*H_3:_ Perceived risk mediates the relation between technology awareness and behavioral intention to use cryptocurrency in Pakistan.*

### Moderating Role of Government Support

Government support is a legal framework developed to regulate that service providers perform their obligations under the set legal framework and guarantee the consumers from online scams, fraud, and violations (Khan et al., 2020). The same is the case for blockchain-based cryptocurrency; government support is mandatory to reduce the uncertainty of its adoption (San Martín and Camarero, 2009). Government instructions and regulations can impact customers’ behavioral intention toward adopting new technology. Government support moderates the consumer’s behavioral intention to use cryptocurrency (Alaklabi and Kang, 2021). It is necessary to facilitate and control the adaptation of new technology even though we know that using technology could benefit and enhance productivity (McCloskey, 2007). Government support can moderate the relationship between technology awareness and consumers’ behavioral intention even though the customers do not understand and comprehend the complexity and use of new technology. Still, because of legal security, they will understand and adopt it (Granić and Marangunić, 2019).

*H_4a:_ Government support moderates the relation of technology awareness on behavioral intention to use cryptocurrency in Pakistan, through perceived usefulness might be more assertive when there is higher government support.*

The government’s support changes the customer behavior toward cryptocurrency, even though the consumers found that it is an easy and effortless system for a medium of exchange (San Martín and Camarero, 2009). It may help to increase acceptability and confidence in adopting new technologies (Uematsu and Tanaka, 2019). The behavioral intention of consumers would be enhanced when supported by the government as it would reduce risk. Customers’ willingness to accept technology ensures that consumers would adopt it if its use were influenced by government support and directions (Khan et al., 2020), as government support plays a significant role in adopting cryptocurrency. It can moderate the relationship between technology awareness and behavioral intention, reducing the degree of uncertainty and the potential adverse effects of utilizing technology even if they do not comprehend and understand it because of legal security. They will put effort into understanding and adopting it. It comprehends the development of this hypothesis:

*H_4b:_ Government support moderates the relation of technology awareness on behavioral intention to use cryptocurrency in Pakistan, through perceived ease of use might be more assertive when there is higher government support.*

Government support, guidelines, and policies are vital to reducing the risk of cryptocurrency adoption as a medium of exchange (Albayati et al., 2020). Previous research highlights that the perceived risk associated with cryptocurrency is high, as it is decentralized in nature. Still, if the government supports it and creates a legal framework, consumers’ behavioral intentions will change toward this new medium of exchange (Mutahar et al., 2018). Risk is the primary factor that directly impacts the technology awareness and behavioral intention to use digital currency as a medium of exchange. Still, if government support is present, it can moderate the effect positively as consumers trust more legality and security (Priem, 2021). Previous research also supports this relationship, so the following hypothesis is constructed:

*H_4c:_ Government support moderates the relation of technology awareness on behavioral intention to use cryptocurrency in Pakistan, through perceived risk might be more robust when there is higher government support.*

**FIGURE 1:**
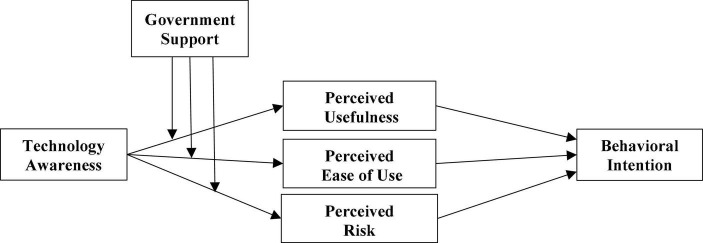
Hypothesized model.

The hypothesized model is presented in Figure 1.

## Materials and Methods

### Participants and Procedure

In recent years, cryptocurrency has gained popularity among the general public, causing a paradigm shift in the financial industry from conventional to blockchain-based technology (Alaeddin and Altounjy, 2018). However, its technical nature depends on internet usage (Csobanka, 2016), which is more common among young people who can quickly connect with new technologies (Fagnant and Kockelman, 2015). Their acceptance of technological advancement is high, especially in terms of financial transactions (Blocki and Zhou, 2016). That is why the current study targeted the Z generation, who are digital natives born between the late 90s (Zafar et al., 2021). So, they are generally more aware of internet usage and display a high degree of technical competency in operating digital tools (Turner, 2015). Therefore, scholars believe they can rapidly evolve into information consumers and suppliers with advanced technological abilities (Scholz and Vyugina, 2019).

Data were collected through a questionnaire that was adapted from relevant literature. The questionnaire was divided into two parts. The first part of the survey included three pre-screening questions to identify the targeted sample participants. These questions were related to age, internet usage, and knowledge of smart tools. If a participant fulfills the criteria, they would be eligible to participate in the survey. 550 questionnaires were distributed to different universities in Lahore *via* Google forums and hard copies. They were informed about the study objective and assured to maintain the confidentiality of their responses. Most respondents were aware of digital currency (83.2%). Almost (99%) of respondents can operate digital tools and spend more than 3 h on the internet daily. According to researchers, 67% of generation Z spent more than 3 h per day using computers and the internet (Hoxha and Zeqiraj, 2020).

Furthermore, data were separated for outliers and unengaged responses, i.e., 39%. The final data consisted of 333 responses from generation Z, who have adequate knowledge of cryptocurrency features and usage. Table 1 shows that the final sample comprises 153 (46%) male and 180 (54%) female respondents. More than half of the respondents (72%) fall under the 18–24 age category. Most of the respondents (83%) were unmarried. The highest category of the respondents has obtained a bachelor’s degree (48%). A total of 33% of the study participants are self-employed or doing jobs in the public or private sector.

**TABLE 1:** Demographic profile.

Demographics category	(*n* = 333)	
	Frequency	Percentage%
**Gender**		
Male	153	46
Female	180	54
**Marital status**		
Married	55	17
Unmarried	278	83
**Age**		
18–24 years old	240	72
25–34 years old	93	28
**Education background**		
High school	51	15
Diploma	3	1
Bachelor’s degree	159	48
Master’s degree	116	35
Ph.D.	4	1
**Occupation**		
Student	224	67
Government employee	23	7
Private sector employee	44	13
Business owner	42	13

### Measures

Technology Awareness is core to technology diffusion. A person can perceive and comprehend the utility of any technology that is becoming popular and widely recognized in business or the market (Almuraqab, 2020). It is calculated by nine items used by Albayati et al. (2020). Perceived usefulness reflects a person’s belief that adopting new technology can be beneficial for them in enhancing their productivity. It was assessed by using six items adapted from Uematsu and Tanaka (2019). Perceived ease of use depicts the individuals’ understanding of new, simple, and accessible technology to adopt. It was measured by six questions borrowed from Saadé and Bahli (2005). Perceived risk refers to consumers’ impression of the degree of uncertainty of utilizing technology because they do not comprehend and understand it (Lim, 2003), measured by three items adapted from Shahzad et al. (2018). Government support includes the legal mechanisms established for the authorized bodies to oversee and ensure network operators and technology users perform their responsibilities and avoid violations (Shafieizadeh et al., 2021). Its moderating role was tested using four questions adapted from Albayati et al. (2020). Behavioral intention is the user’s anticipated possibility that leads them toward a specific behavioral outcome (Yi et al., 2021). It helps customers accept useful new technology and understand its importance before altogether rejecting it (Yuen et al., 2021). It was measured by using five items taken from Treiblmaier and Sillaber (2021). All items were assessed by using a 5-point Likert scale. The internal consistency and reliability of the scale also lie within an acceptable range for all the items, as the factor loadings range from 0.749 to 0.895 (see Table 2).

**TABLE 2:** Measurement items and standardized factor loadings.

Constructs	SFL
*Behavioral intention (BI)*α = *0.897; CR* = *0.924; AVE* = *0.708*	
BI1	0.831
BI2	0.839
BI3	0.859
BI4	0.841
BI5	0.838
*Government support (GS)*α = *0.850; CR* = *0.898; AVE* = *0.639*	
GS1	0.784
GS2	0.782
GS3	0.874
GS4	0.875
*Perceived ease of use (PEOU)*α = *0.908; CR* = *0.929; AVE* = *0.687*	
PEOU1	0.846
PEOU2	0.850
PEOU3	0.817
PEOU4	0.844
PEOU5	0.860
PEOU6	0.751
*Perceived risk (PR)*α = *0.840; CR* = *0.903; AVE* = *0.757*	
PR1	0.873
PR2	0.852
PR3	0.884
*Perceived usefulness (PU)*α = *0.925; CR* = *0.941; AVE* = *0.727*	
PU1	0.801
PU2	0.842
PU3	0.847
PU4	0.849
PU5	0.895
PU6	0.880
*Technology awareness (TA)*α = *0.943; CR* = *0.952; AVE* = *0.689*	
TA1	0.839
TA2	0.849
TA3	0.863
TA4	0.864
TA5	0.845
TA6	0.782
TA7	0.859
TA8	0.817
TA9	0.749

## Results

Table 3 shows discriminant validity results by employing the Fornell–Larker criteria. It explains how many constructs are unrelated to each other in the model, i.e., constructs differ from each other (Shahzad et al., 2021). It is accessed by evaluating cross-loading values in two ways: the Fornell–Larcker criterion and the other is the Hetrotrait–Monotrait ratio (HTMT). However, for measuring discriminant validity, the self-loading values of each construct should be greater than other construct values (Khan et al., 2021). As shown in Table 3, all the bold diagonal values are different from each other, and the AVE square roots correlated more than the standardized correlation. Further, the structural model is run on PLS-SEM, and the model fitness is also checked. The statistical values of (SRMR = 0.05; χ^2^ = 2229.91; NFI = 0.70) reflect that the overall model is a good fit.

**TABLE 3:** Inter-construct correlation and discriminant validity.

Variables	Mean	SD	BI	GS	PEOU	PR	PU	TA
BI	3.985	1.012	0.842					
GS	3.834	1.239	0.147	0.830				
PEOU	4.048	1.012	0.084	0.568	0.829			
PR	4.340	1.044	0.778	0.230	0.177	0.870		
PU	4.048	1.012	0.821	0.266	0.235	0.806	0.853	
TA	4.151	1.174	0.132	0.557	0.682	0.257	0.300	0.830

The R-square values for perceived factors (usefulness, ease of use, risk) and behavioral intention are (0.14, 0.53, 0.11) and 0.69, respectively, showing that independent variables explain a significant part of the variance. Table 3 shows that all the diagonal values of the construct are greater than the off-diagonal values, which explains that all the values are in an acceptable range and the data is valid. The mean value for behavioral intention (3.985), government support (3.834), perceived use (4.048), perceived usefulness (4.048), perceived risk (4.340), and technology awareness (4.151) is high. At the same time, the values of standard deviation show the normality in data for all the variables, which depicts less dispersion in the data.

We used SEM to test the hypothesis in the current study. The mediating effects of perceived factors (usefulness, ease of use, risk) are calculated using the bootstrapping method. Table 4 shows the results of hypothesis testing. The SEM findings show direct effects and indirect effects on the paths. Technology awareness directly relates to perceived factors (usefulness, ease of use, risk). They all correlate to each other, and results show their significance, as beta value, *t*-value, and *p-*values are on threshold (β = 0.283, 0.482, 0.245; *p* = 0.001, 0.000, 0.00), respectively. The research further reveals that perceived factors (usefulness, ease of use, risk) directly relate to behavioral intention. All the values are significant for the path model (β = 0.171, 0.691, *p* = 0.011, 0.000), respectively. Thus, all the hypotheses were accepted.

**TABLE 4:** Results for structural equation model.

Hypothesized path	Beta	Mean	*SD*	*T*-values	*p*-values	Result
i. Direct effect						
TA- > PU	0.283	0.285	0.057	4.958	0.000	Supported
TA- > PEOU	0.482	0.476	0.057	8.406	0.000	Supported
TA- > PR	0.245	0.246	0.056	4.356	0.000	Supported
PU- > BI	0.691	0.686	0.073	9.821	0.000	Supported
PEOU- > BI	0.108	0.103	0.042	2.595	0.010	Supported
PR - > BI	0.171	0.179	0.067	2.559	0.011	Supported
ii. Indirect effects						
TA - > PU - > BI	0.195	0.194	0.039	5.007	0.039	Supported
TA - > PEOU - > BI	0.052	0.049	0.021	2.473	0.014	Supported
TA - > PR - > BI	0.042	0.044	0.021	2.024	0.044	Supported
iii. Moderating effect						
TA*GS- > PU- > BI	0.109	0.114	0.044	2.472	0.014	Supported
TA*GS - > PEOU- > BI	0.013	0.013	0.007	1.969	0.049	Supported
TA*GS - > PR- > BI	0.033	0.035	0.016	2.074	0.039	Supported

The study also analyzed the mediating role of perceived factors (usefulness, ease of use, risk) associated with technology awareness and intention to use digital currency. The results show that perceived usefulness is a mediator between technology awareness and behavioral intention to adopt cryptocurrency in Pakistan; the results are significant (β = 0.195, *p* = 0.039). Perceived ease of use also has a significant impact as a mediator on technology awareness and behavioral intention to adopt cryptocurrency in Pakistan. The results show the significance of this path (β = 0.052, *p* = 0.014). Perceived risk is also a mediator and depicts significant results (β = 0.042, *p* = 0.044). Thus, these results show that perceived factors (usefulness, ease of use, risk) mediated the relationship between technology awareness and behavioral intention to adopt digital currency in Pakistan. Hence, all the hypotheses are accepted H_1_, H_2_, and H_3_.

Additionally, in Table 4, we calculated the role of government support as a moderator with the path (technology awareness*government support- > perceived usefulness- > behavioral intention), and the values observed are (β = 0.109, *p* = 0.014), which are highly significant; thus, H_4a_ is accepted. The second moderating effect of government support (technology awareness*government support- > perceived ease of use- > behavioral intention) moderates the relationship between technology awareness and perceived ease of use on behavioral intention, as the results are highly significant (β = 0.013, *p* = 0.049). So, H_4b_ is accepted. The third moderating effect of government support (technology awareness*government support- > perceived risk- > behavioral intention) is between perceived risk and technology awareness shows (β = 0.025, *p* = 0.020). All the values are highly significant; hence, H_4c_ is also accepted.

**FIGURE 2:**
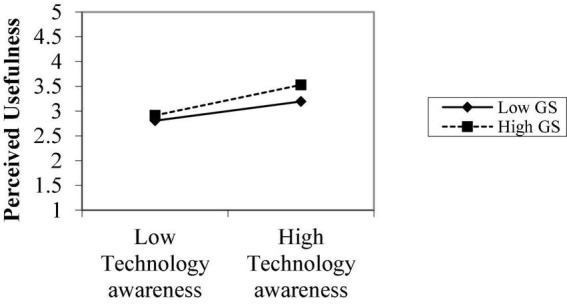
Moderating role perceived usefulness.

**FIGURE 3:**
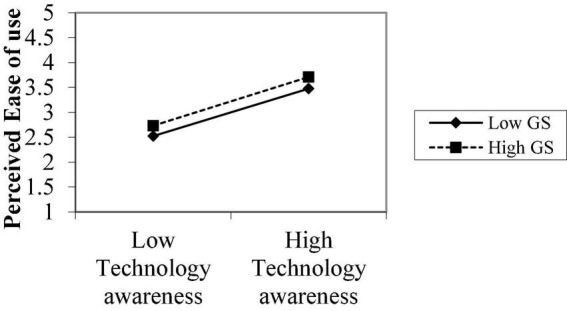
Moderating role perceived ease of use.

**FIGURE 4:**
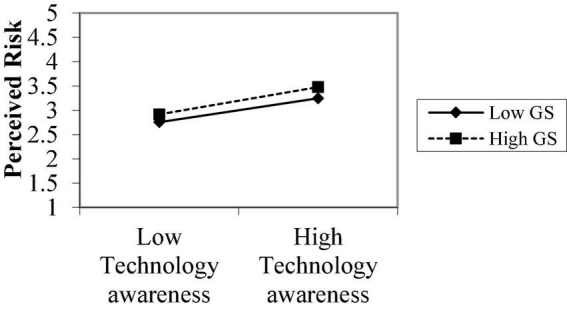
Moderating role perceived risk.

We plotted the graphs to explain the results of moderating the role of government support. Figures 2–4 show the moderating role of government support between perceived ease of use, perceived usefulness, and perceived risk, respectively. Figure 2 explains the mediating role of government support between technological awareness and perceived usefulness. The notable point on the moderation graphs is perceived usefulness and low level of government support. The graphs show that technological awareness and perceived usefulness are stronger under high government support. This eventually supports H_4a_.

Figure 3 explains the moderating role of government support between technological awareness and perceived ease of use. The notable point on the moderation graphs is perceived by the ease of use and low level of government support. The graphs show that technological awareness and perceived ease of use are stronger under high government support, supporting H_4b_. Figure 4 explains the moderating role of government support between technological awareness and perceived risk. The notable point on the moderation graphs is perceived risk and low level of government support. The graphs show the relationship between technology awareness and risk is stronger under high government support, supporting H_4c_.

## Discussion

The internet has become a convenient mode of information in the twenty-first century, which has changed the monetary system worldwide and the transaction mode from cash to the online system (Almuraqab, 2020). The technological advancement of personal computers, mobile phones, and tablets influenced the trend of online transactions through digital currencies (Yi et al., 2021). As a result, cryptocurrency gained popularity and has drastically transformed the international and national financial markets. The present study evaluates the factors that influence the adaptability of cryptocurrency in Pakistan. The study investigated the impact of technological awareness on consumer behavioral intention to adopt cryptocurrency through the perceived factors (usefulness, ease of use, risk). It also investigates the moderating role of government support between the indirect relationships of technology awareness and behavioral intention. The underlying conceptual framework is empirically tested by surveying 333 participants from generation Z. The study has three significant findings.

First, the results indicated that technological awareness positively impacts behavioral intention. Higher technology awareness increases consumers’ confidence in adopting new technologies (Kliestik et al., 2020). Prior studies show the same results by explaining the main elements that affect consumer behavioral intention in adopting the technology: awareness and technical knowledge (Irfan et al., 2022). Second, it investigated the mediating role of perceived predictors (usefulness, risk, ease of use) on behavioral intention to adopt cryptocurrency in Pakistan. The results show that the relation between technology awareness and behavioral intention is mediated by perceived usefulness, which is confirmed by the prior literature in the context of TAM (Anser et al., 2020).

Perceived ease of use also mediated the relationship between technology awareness and consumers’ behavioral intention. It enhances consumers’ confidence in adopting new technology, believing it can benefit them (Johar et al., 2021). Consumers have higher technology awareness and believe that it will increase their performance and be easy to adopt. This will changes their perception and increase the behavioral intention to accept new technology (Albayati et al., 2020). The relationship between technology awareness and behavioral intention to use cryptocurrency in Pakistan is mediated by perceived ease of use, emphasizing making a user-friendly system, i.e., easy to run, simple, responsive, and adaptable. Furthermore, perceived risk can also mediate the relation between technology awareness and behavioral intention as it is a consumer’s perception that varies in different consequences of adopting technology. Cryptocurrencies are considered riskier as any central body does not regulate them. Higher technical knowledge and awareness can minimize perceived risk and mediate the positive relationship between technology awareness and consumers’ behavioral intention (Jalan et al., 2021).

Third, it analyzes the moderating role of government support between direct and indirect relationships of the structural path model of the hypotheses. Government support moderates the relation of technology awareness to behavioral intention through perceived usefulness that might be more assertive when there is strong government support (Chen and Aklikokou, 2020). Government support includes the legal mechanisms established for the regulatory bodies to oversee and guarantee that network operators and technology users perform their responsibilities and avoid violations. The adaptability of cryptocurrency can be enhanced if the government enforces legal regulations to control and prevent fraud for the use of blockchain-based applications through a clear understanding of the transaction procedure. The study results show that it has a positive association among them. Perceived ease of use more positively mediates when the government support is higher because consumers feel safe with rules and regulations (Tandon et al., 2021). Finally, perceived risk can also positively mediate the behavioral intention of consumers if the government support is higher, as it can reduce risk in cryptocurrency adoption. The main hindrance to its adoption is that it is non-regulated, and consumers feel riskier investing in it (Bin, 2022). Finally, we conclude that higher government support positively impacts the relationship between technology awareness and consumers’ behavioral intention through perceived factors (usefulness, risk, ease of use).

## Conclusion

Cryptocurrency is attracting the general masses and gaining the attention of consumers, investors, investment industries, and regulators, but its acceptance among these stakeholders is still questionable. The current study sheds light on factors affecting the adaptability of cryptocurrency in Pakistan with an integrated TAM. This study has three significant findings. First, it reveals that technological awareness positively impacts consumers’ behavioral intention to adopt it. Cryptocurrency usage is limited in developing economies due to a lack of technological awareness. Therefore, enhancing the technological level and explaining a clear grasp of each transaction procedure can positively influence the behavioral intention of crypto users to adopt it as a medium of exchange. Second, it explains the intervening role of perceived factors (usefulness, risk, ease of use) between technology awareness and behavioral intentions. Third, it concludes that government support significantly impacts the relationship between technology awareness and behavioral intention directly and also through the indirect path of perceived factors, such that the effect of technology awareness on behavioral intention through perceived factors (usefulness, ease of use, risk) is more assertive when government support is high. The findings underscore the importance of the TAM in influencing customers’ behavioral intentions, decisions, and acceptance of cryptocurrency.

### Implications of the Study

#### Theoretical Implications

This study aims to determine the predictors of the cryptocurrency acceptance model by investors, handlers, and/or customers, allowing current and prospective market participants to analyze its essential features. It explains the mediating role of perceived factors (usefulness, ease of use, risk) between technology awareness and behavioral intentions. In this way, it adds to the existing body of knowledge discussing the predictors for adopting cryptocurrency. Further, it highlights the intervening role of government support through moderated mediation model, which can help scholars to understand the importance of legal regulations and government support in enhancing users’ behavioral intentions. The current and prospective investors believe that blockchain-based applications are safe when regulated and supported by the government. Therefore, government support reflects the behavioral intention of the consumer and serves as a trust mechanism that can increase the confidence of investors to use blockchain-based cryptocurrency.

#### Practical Implications

The present study has provided significant insight into the development of blockchain technology as the medium of exchange in emerging economies like Pakistan. Pakistan is a developing nation. Its population is approximately 220 million. Although the literacy rate has been steadily increasing, still it lacks an understanding of digital development due to poor literacy and internet usage (Zafar et al., 2021). As a result, today, many non-tech savvy citizens are unaware of digital advances and businesses (Irfan et al., 2022). The findings of the current study will help the government pay special attention to this knowledge area and educate the masses regarding the new advancements in the field. Secondly, the rapid change in the worldwide monetary and financial systems enhances the need to develop, modify and make policies to respond to the emerging virtual currencies. But in many countries, including Pakistan, there is no regulatory framework available to support crypto-transactions. The current study highlights the need to develop a policy framework that can help to monitor crypto-based transactions. Thirdly, cryptocurrency is a fast, decentralized, and borderless means of payment by restoring the usual fiat money. According to an estimation, 9 million people in Pakistan, who account for 4.1% of the total population, own cryptocurrencies. There may be chances of illegal money transfers or financial fraud in crypto-based transactions. Therefore, policymakers design the criteria that can deduct the online scams and illegal transfer of money through a centralized monetary system.

### Limitations and Future Recommendations

Despite significant contributions, this study faces certain limitations that allow future research to investigate the underlying area. Firstly, it focuses on the Z generation, and the sample mainly consists of graduates and highly educated individuals whose behavior may differ from the general population. They are mostly inventive by nature and can quickly interact with new technologies, which may introduce biases into the main findings. Further, the Z generation is less likely to be aware of the implications and repercussions of digital currency use. Future research can be conducted on another population sample. Second, in this research, technology awareness is used as the primary independent variable; for future research, other variables like social influence, trust, and design can further be tested with this integrated TAM. Third, the research is limited to geographical boundaries. Data were collected just from one city. For future work, samples can be collected from different major cities. Fourth, the present research shows the adaptability of digital currency for Pakistan. Future research can be conducted by comparing the adaptability of cryptocurrency globally. Finally, the research is limited to factors that influence cryptocurrency adoption. Future research should look at the long-term viability of cryptocurrencies and bitcoin mining.

## Data Availability Statement

The raw data supporting the conclusions of this article will be made available by the authors, without undue reservation.

## Author Contributions

NS contributed to the conceptualization and writing of the initial draft. KK contributed to visualizing, supervising, and finalizing the research. SF helped in data collection. SM entered the data and performed analysis. TR and HJ helped in the review process. All authors proofread the final version and approved the submitted version.

## Conflict of Interest

The authors declare that the research was conducted in the absence of any commercial or financial relationships that could be construed as a potential conflict of interest.

## Publisher’s Note

All claims expressed in this article are solely those of the authors and do not necessarily represent those of their affiliated organizations, or those of the publisher, the editors and the reviewers. Any product that may be evaluated in this article, or claim that may be made by its manufacturer, is not guaranteed or endorsed by the publisher.
